# Environmental factors influencing fungal growth on gypsum boards and their structural biodeterioration: A university campus case study

**DOI:** 10.1371/journal.pone.0220556

**Published:** 2019-08-02

**Authors:** Negin Kazemian, Sepideh Pakpour, Abbas S. Milani, John Klironomos

**Affiliations:** 1 Department of Biology, University of British Columbia, Kelowna, Canada; 2 School of Engineering, University of British Columbia, Kelowna, Canada; 3 Infectious Disease and Microbiome Program, Broad Institute, Cambridge, Massachusetts, United States of America; 4 Composites Research Network, Okanagan Node, Kelowna, Canada; Cardiff University, UNITED KINGDOM

## Abstract

The new era in the design of modern healthy buildings necessitates multidisciplinary research efforts that link principles of engineering and material sciences with those of building biology, in order to better comprehend and apply underlying interactions among design criteria. As part of this effort, there have been an array of studies in relation to the effects of building characteristics on indoor microbiota and their propensity to cause health issues. Despite the abundance of scientific inquiries, limited studies have been dedicated to concomitantly link these effects to the deterioration of ‘structural integrity’ in the building materials. This study focuses on the observed biodeteriorative capabilities of indoor fungi upon the ubiquitous gypsum board material as a function of building age and room functionality within a university campus. We observed that the fungal growth significantly affected the physical (weight loss) and mechanical (tensile strength) properties of moisture-exposed gypsum board samples; in some cases, tensile strength and weight decreased by more than 80%. Such intertwined associations between the biodeterioration of building material properties due to viable indoor fungi, and as a function of building characteristics, would suggest a critical need towards multi-criteria design and optimization of next-generation healthy buildings. Next to structural integrity measures, with a better understanding of what factors and environmental conditions trigger fungal growth in built environment materials, we can also optimize the design of indoor living spaces, cleaning strategies, as well as emergency management measures during probable events such as flooding or water damage.

## Introduction

Indoor environments, as the main habitats of modern humans, encompass a complex mixture of viable and dead microorganisms [1] which can affect occupants' health and also deteriorate different parts of buildings. This may also lead to undesirable changes in the structural properties of the building materials [2–6]. Among different types of indoor microbiota, the fungal mould growth and associated biodeterioration during the service life of buildings continue to be a major concern for architects, structural engineers, emergency management teams, and healthcare authorities. Mould growth is a housing epidemic across Canada with 13% of households in 2009 having reported their presence [7]. It is a particular problem for the populations living on the First Nations reserves in Canada. For example, mould was found in 69% of homes on a reserve in the central coastal area of Vancouver Island [8]. The biodeteriorative effect of these organisms on building materials can itself lead to major economic impacts across the world with substantial renovation, replacement, and remedial measure costs. In 1977, the United Kingdom (UK) estimated that the cost of repairing fungal-damaged timber used in construction amounted to €3 million per week [9]. The cost estimates for the replacement of decaying wood consumes 10% of the timber cut annually in the US and amounted to $613 million in 1988, which did not consider the costs of replacement, liability, and preventative treatment [10]. Mould growth on other types of materials such as gypsum boards has similarly led to removal costs of $485–590 for a 9.3 m^2^ area [11] and replacement costs of $480–720 for rooms requiring 12 panels of gypsum board building material [12]. Finally, substantial amounts of mould growth have occasionally led to the collapse of buildings, such as the balcony collapse of Berkeley in 2015, which tragically led to the injury of seven and death of six individuals [13].

In parallel, the health effects of mould growth and its exposure is another problematic issue facing occupants of built environments. Metabolites produced by fungi such as mycotoxins may cause a toxic response at a low dosage [2] and can be absorbed from the skin, airways, and intestinal lining [14]. Potential hazardous fungi associated with mycotoxin production include *Aspergillus versicolor* (sterigmatocystin), *Aspergillus fumigatus* (gliotoxin), *Aspergillus niger* (ochratoxins), *Alternaria alternata* (tenuazonic acid), and *Stachybotrys chartarum* (trichothecenes) [2, 15, 16], which can lead to multisystemic effects such as gastrointestinal, cardiovascular, and neuropsychiatric complications [17–19]. However, further studies are required in order to transition from association to causation. Potential health complications associated with poor indoor air quality and fungal exposure can also have financial consequences [20]. Recently, it was shown that, of the 21.8 million people that were reported to have asthma in the USA, approximately 4.6 million cases were attributable to dampness and mould exposure in the home, with an estimated economic damage of $3.5 billion [21]. It is worth noting that dampness can also support the growth of dust mites and actinomycetes, which can lead to challenges in the isolation of health effects solely due to fungal exposure [22].

“How indoor fungal communities assemble” has been the focus of numerous recent studies. The most recent results suggest that indoor fungal assemblages (dead, dormant, and viable fungal species) are a random subsample of outdoor fungi and that a major determinant of the composition is the dispersal of species from outdoor sources [23]. These organisms can grow on organic and inorganic substrates [24] and are categorized based on their water activity including primary colonizers (growth at low moisture level), secondary colonizers (growth at intermediate moisture level), and tertiary colonizers (growth at high moisture level) [15]. Indoor environmental variables such as building materials can also select for different fungal communities (viable fungal species) [25–29], thus building material can vary in the type of fungal growth that they can support. Many of the construction and building materials, made up of natural and manmade compounds, contain natural organic polymers including starch, cellulose, hemicellulose, pectin, and lignin which are susceptible to fungal growth [30]. For example, wood (another commonly used indoor material) favours the growth of numerous species from different genera including *Penicillium*, *Aspergillus*, *Aureobasidium*, *Trichoderma*, *Cladosporium*, *Chaetomium*, *Alternaria*, *Eurotium*, and *Acremonium* [25–28], but not *A*. *versicolor*, *Calcarisporium arbuscula*, and *Sporothrix* spp. [27]. Variation within the same material products is also evident [31]. For instance, oriented strand board (OSB) plywood and medium density fiberboard (MDF) are more susceptible to the growth of *Aspergillus*, *Trichoderma*, and *Penicillium* spp. [31], while wood types such as Douglas-fir heartwood are less susceptible to these microorganisms [32]. Inorganic compounds can also support mould growth due to dust absorption such as in the case of fiberglass insulation of ceilings [26, 29, 33, 34]. Types of flooring material, carpets in particular, have become another concern in the design of indoor spaces as they accumulate dirt and debris and can be associated with fungal growth [35, 36]. The commonly used synthetic polymers in these materials can also suffer from degradation [1].

Gypsum boards, commonly known as ‘drywall’ and invented in 1894 [37], are one of the most popular building materials that are made up of ‘both organic and inorganic’ components. This indoor material is made up of a gypsum plaster core composed of a naturally occurring mineral, calcium sulfate dihydrate (CaSO_4_·2H_2_O), which is sandwiched between two thick sheets of paper [38, 39]. Although the cellulosic paper is the main factor leading to vulnerable indoor materials susceptible to fungal growth [34], the gypsum itself can also support growth due to its nutrient content and additives [27]. Other factors affecting the growth of these microorganisms on this indoor substrate include the presence of water as the limiting factor [25, 40, 41] in conjunction with the material content and characteristics (e.g., level of alkalinity [42], porosity [43], density [44], and the presence of biocides [45, 46]). Alongside the microbial driven biodeteriorative mechanisms, sufficient water and humidity levels, as well as abiotic factors such as temperature, photodegradation, and insects can also affect the structural integrity of materials [47]. Dampness in buildings is often associated with simultaneous factors including visible water damage or stains, visible mould, odours [22], as well as biodeterioration of building materials and structures [48]. It has been reported that gypsum boards are highly associated with fungal growth from numerous species of *Stachybotrys*, *Penicillium*, *Acremonium*, *Chaetomium*, *Trichoderma*, and *Aspergillus* genera [25, 26, 33]. Furthermore, a study by Andersen et al. [49], showed that *Neosartorya hiratsukae*, *Chaetomium globosum*, and *S*. *chartarum* were the dominant fungal species found on gypsum boards, but interestingly they were already incorporated into the material during production. Understanding the control factors influencing the fungal communities that are able to grow on this integral building material is deemed critical since preventative measures can cease the biodeteriorative abilities of these microorganisms and avoid costly remediations.

In addition, the association of the mechanical deterioration of building materials (here gypsum boards) with viable indoor fungal taxa, as triggered by different environmental selective pressures, is yet to be understood. Accordingly, the overall scope of this case study, conducted at a university campus, was to better understand the different environmental conditions that can lead to mould growth on a commercial gypsum board type and to link the identified fungal community composition of indoor dust to the mechanical and physical properties of the drywall samples, under a hypothetical scenario of water-damage/flooding. The specific questions of the study were: (1) Would different building characteristics/environmental variables (such as the age of building, different types of rooms, flooring type, temperature, humidity, occupancy level, general cleanliness) affect the diversity and abundance of the fungal community able to grow on the gypsum board? (2) If yes, are there health-risk fungi that would grow under particular building characteristics? (3) To which extent can the above differences in building characteristics and the associated fungal growths alter the biodeterioration of both physical and mechanical properties of the gypsum board?

## Materials and methods

### Dust sample collection and environmental assessment

Sample collection was conducted at the University of British Columbia-Okanagan Campus, Kelowna, Canada. The design of experiment consisted of three main controlled factors including (i) age of the building, (ii) type of room within the building (office, classroom, laboratory), and (iii) type of flooring (with or without carpet) (Table 1). Other uncontrolled factors including room temperature, humidity, occupancy level, and cleanliness level, were also monitored and included in the analysis (as uncontrolled factors or random effects). Indoor Solar Powered Wireless Sensor (model WS-6020U-IT, La Cross Technology, USA) was used for indoor temperature and humidity measurements. Occupancy level was defined as low (<10), medium (10–20), and high (>20), while general dustiness scoring was defined as 1: very clean, 2: moderate, and 3: unclean.

**Table 1:** Dust sample collection design.

Building Name	Year Built	Construction Type	No. of Floors	Presence of Atrium	Building age	Office		Laboratory		Classroom	
						Carpet	No Carpet	Carpet	No Carpet	Carpet	No Carpet
**EME**^a^	2011	Concrete	5	No	New	3	0	0	3	0	3
**ASC**^a^	2010	Concrete	4	No	New	3	3	0	3	2	3
**ARTS**^a^	1992	Steel frame	3	Yes (enclosed)	Old	3	3	0	3	3	2
**SCI**^a^	1992	Steel frame	3	Yes (open)	Old	3	2	0	3	3	3

Dust sample collection design, including three controlled factors of (i) age of building (new: EME and ASC, and old: ARTS and SCI), (ii) type of room within each building (office, classroom, laboratory), and (iii) type of flooring (carpet versus no carpet). Note that two samples were collected in each room in new buildings (Total = 23 rooms), old buildings (Total = 28 rooms), classrooms (Total = 19 rooms), laboratories (Total = 12 rooms), offices (Total = 20 rooms), carpeted rooms (Total = 20 rooms), and non-carpeted rooms (Total = 31 rooms); i.e. a total of 51 rooms.

^a^ Name of buildings: Engineering/Management/ Education (EME), Arts and Sciences Centre (ASC), Arts (ARTS), and Science (SCI).

Dust samples were collected from a total of 51 rooms (two repeats per room; Table 1) using a passive petri plate gravitational dust settling method [50]. Empty (growth-medium-free) polystyrene petri dishes were set up 2.5 m above ground level in each selected environment for a duration of one month.

### Material sample preparation and biodeterioration assessment

Under aseptic conditions, the collected dust from each room (after one month of exposure) in each petri dish was extracted using 125 ml of sterilized aqueous solution with 0.5% Tween 20 (Amresco, Solon, OH, USA). Subsequently, the contents were poured into sterile 500 ml glass bottles (S1 Fig). Commercially available regular gypsum boards (122 cm × 244 cm × 1.3 cm CGC Sheetrock Brand Ultralight Panels) with no modifications or additives were cut into 5 cm × 8 cm pieces. The gypsum boards were exposed to ultraviolet radiation for 24 hours (turned to expose all edges) and weighed. Since 2 petri dishes of dust were collected in each room, one was utilized for fungal community and growth coverage assessment (batch 1) and the other for physical and mechanical characterization (batch 2). Batch 1 gypsum boards were placed in the corresponding bottles and submerged in 125 ml of water and dust to assess the fungal growth level on each sample (n = 51). Wet controls with no dust were also submerged in water (n = 5), while dry controls with no dust were kept dry for the duration of the study (n = 5). Similarly, a batch 2 of cut gypsum boards (n = 51) was submerged into water and dust, along with wet controls (n = 10). A set of dry controls was also added (n = 10) prior to physical and mechanical testing. All samples (batch 1 and 2) were left at room temperature for 4 weeks to allow for fungal growth.

### Fungal community and growth coverage assessment

Using batch 1 samples, percent fungal coverage on the gypsum boards were assessed in Image-J [51] with the following scoring scheme:

1 = minimal growth, (growth covering 0–20% of the sample area)
2 = growth covering 20–40% of the sample area
3 = growth covering 40–60% of the sample area
4 = growth covering 60–80% of the sample area
5 = growth covering majority (80–100%) of the sample area

For diversity and fungal community assessments, viable colonies of fungal taxa that were able to grow on the gypsum boards were transferred onto Potato Dextrose Agar (Difco, Detroit, Michigan, USA) and incubated at room temperature for one week. Pure cultures from all morphologically different colonies were sub-isolated for identification, using macroscopic and microscopic characteristics. The gypsum boards were further assessed for three pathogenic fungi that commonly grow on indoor wet materials: *A*. *alternata*, *A*. *versicolor*, and *A*. *fumigatus* [16, 25, 52]. Total DNA was extracted from 500 mg of fungi growing on each of the gypsum boards using the FastDNA SPIN Kit for Soil (MP, Biomedicals, LLC, Solon, OH, USA) according to the manufacturer's instructions. PCR amplifications were carried out using species-specific primers for each pathogenic taxon (Table 2), confirmed by Sanger sequencing. Positive controls for each target taxa are described in Table 2. A negative control, consisting of the reaction mixture without DNA, was also used in each PCR run.

**Table 2:** PCR primers utilized.

Fungal species	Primer name	Primer sequence (5'—3')	Annealing temperature (°C)	Amplicon size (bp)	Positive control
*Aspergillus fumigatus* [53]	AfumiF1 AfumiR1	GCCCGCCGTTTCGAC CCGTTGTTGAAAGTTTTAACTGATTAC	50	136	ATCC 34506^a^
*Aspergillus versicolor* [53]	AversF2 AversR1	CGGCGGGGAGCCCT CCATTGTTGAAAGTTTTGACTGATCTTA	50	109	ATCC 44408^a^
*Alternaria alternata* [53]	AaltrF1 AltrR1	GGCGGGCTGGAACCTC GCAATTACAAAAGGTTTATGTTTGTCGTA	52	123	PEM 01043^b^

PCR primers used to identify the health hazardous indoor fungi on gypsum board specimens.

^a^ American Type Culture Collection, VA, USA

^b^ Prestige EnviroMicrobiology Inc., NJ, USA

PCR mixture (50 μl) contained 10 μl of 5X Green Go Taq Flexi Buffer, 200 μM dNTPs, 2 μl MgCl_2_ (25 mM), 0.2 μM of each primer, 1.25 U of Go Taq G2 Hot Start Polymerase (Promega, Madison, WI, USA), nuclease-free water (IDT-Coralville, IA, USA), and 2 μl of extracted DNA (5 ng/μl). The PCR conditions started with an initial DNA denaturation (94°C for 2 min), followed by 30 cycles of 1 min at 94°C (denaturing), 1 min of annealing at temperatures specified in Table 2, and 1 min at 72°C (extension), followed by a final extension of 5 min at 72°C. The size (Table 2) and specificity (unique band) of PCR products were then determined by comparison with DNA standards (1kb DNA Ladder, Invitrogen, CA, USA) after agarose gel electrophoresis.

#### Mechanical biodeterioration assessment

Batch 2 specimens were randomly divided into two groups for (a) physical property testing: (dry control; n = 5), (wet control; n = 5), (samples with varying ranges of % growth coverage; n = 25), and (b) mechanical property testing (dry control; n = 5), (wet control; n = 5), and (samples with varying ranges of % growth coverage; n = 21). Group (a) specimens were drained of excess water and placed in an oven at 50°C for one week to measure their dry weights. For tensile testing (group (b)), the gypsum boards were air-dried, the paper backing was separated, and cut in half vertically. The tensile tests were performed using the Instron 5969 machine and the strength property of material samples was measured under a modified ASTM D828-97 standard. The modification included the removal of the paper backing from the gypsum (containing less fungal growth) prior to tensile testing and cutting the samples vertically in two in order to prevent the gypsum from dominating the paper tensile strength. For microstructural visualization purposes, selected gypsum board papers with varying ranges of % growth coverage (n = 10), as well as dry (n = 3) and wet controls (n = 3), were tested using a Tescan Mira3 XMU Field Emission Scanning Electron Microscope/SEM (Tescan, Kohoutovice, Czech Republic) on fractured surfaces.

### Statistical analysis

Nested Generalized Linear Model (GzLM; with a multinomial logistic regression as the link function) was used to assess the associations between the controlled environmental factors (age of the building, type of room within the building, and type of flooring) and the fungal growth coverage and fungal diversity responses, using SPSS (IBM, USA). The contribution percentage of the controlled parameters was assessed using the maximum likelihood method. For each type of analysis where the normality assumption was not met, the parametric test of ANOVA was used, with a Dunn-Bonferroni post hoc test. The principal component analysis (PCA) [54] correlation was employed to test the direct association between fungal growth coverage level with both physical (weight loss) and mechanical (tensile strength) properties of the gypsum boards. Finally, Spearman correlation analysis was employed to investigate the relationship between fungal growth coverage with physical and mechanical properties.

## Results

### Fungal community and growth coverage assessment

**Fig 1:**
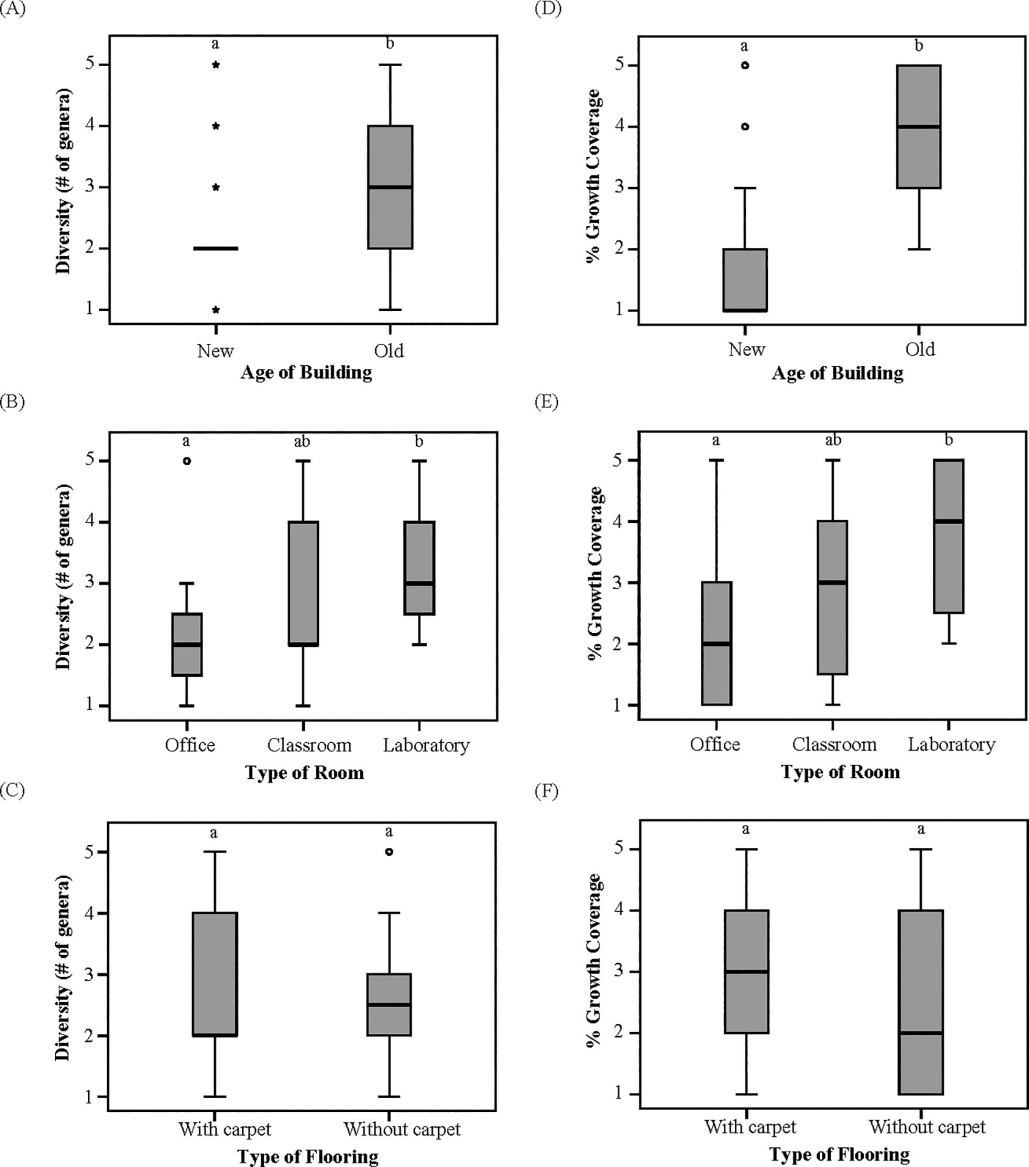
Indoor factors. The relationship between indoor factors of (a) age of building, (b) type of room, and (c) type of flooring, influencing % fungal growth coverage range (1–5) and fungal diversity (# of genera) on gypsum board samples across 51 sampled rooms on campus. Control samples had no fungal growth. Capital letters between boxplots indicate significant differences in % growth coverage and fungal diversity between the factors using GzLM and Dunn-Bonferroni post hoc tests (p<0.05).

**Fig 2:**
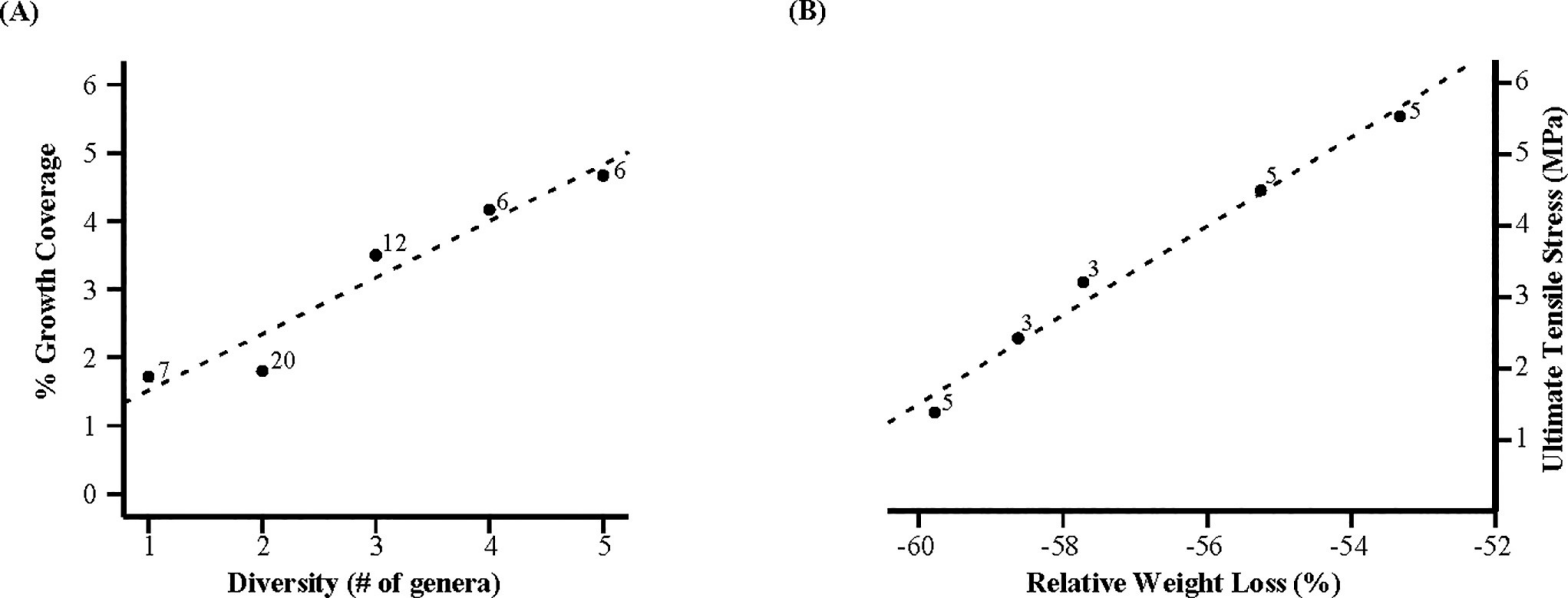
Correlating factors. Visualization of a high correlation between (A) % growth coverage and diversity (# of genera) for the average of all 51 rooms sampled with p<0.01 and R^2^ = 0.932. Each data point represents multiple rooms as displayed with numbers by each point. A high correlation between (B) relative weight loss (%) and ultimate tensile stress (MPa) was observed for the average of all 21 gypsum board samples tested with p<0.01 and R^2^ = 0.984.

Results showed that diverse indoor fungal taxa can grow on gypsum boards in the presence of humidity with *Aspergillus* spp., *Penicillium* spp., and *Cladosporium* spp. being the most common, and *Alternaria* spp., *Stachybotrys* spp., and *Fusarium* spp. being the least common fungi found in indoor environments (Table 3). When considering the tested indoor factors influencing fungal diversity (richness), the age of building (χ^2^(1, N = 51) = 12.37, p = 0.000) and type of room (χ^2^(2, N = 51) = 11.39, p = 0.003) were found to be significantly associated with taxonomic richness, while type of flooring did not (χ^2^(1, N = 51) = 2.70, p = 0.100) (Fig 1). The % growth coverage of fungi was similarly associated with the age of building (χ^2^(1, N = 51) = 50.34, p = 0.000), and type of room (χ^2^(2, N = 51) = 27.44, p = 0.000), while type of flooring had no significant effect (χ^2^(1, N = 51) = 3.05, p = 0.081) (Fig 1). More specifically, the older buildings clearly had a higher fungal diversity (3.1±1.3) than the newer buildings (2.2±0.9) (Fig 1A). The laboratories across the campus had a higher fungal diversity (3.2±0.9) when compared to offices (2.2±0.9) (Fig 1B). No significant differences, however, were observed for the classrooms (2.9±1.4) compared to the laboratories and offices (Fig 1B). Similarly, older buildings led to a higher coverage by fungal growth on the samples as 3.7±1.1 (mean ± SD), while the newer buildings led to a lower amount of fungal growth as 1.7±1.1 (Fig 1C). The laboratories across the campus had a higher % growth coverage (3.7±1.2) when compared to offices (2.1±1.2) (Fig 1D). No significant differences, however, were observed for the classrooms (3±1.6) compared to the laboratories and offices (Fig 1E). The wet control (n = 5), and dry control (n = 5) samples had no observed fungal growth. Overall, the fungal taxonomic richness (# of genera) was positively correlated with % growth coverage of fungi on the gypsum boards (*Rho* = 0.74, n = 51, p<0.01) (Fig 2A).

**Table 3:** Fungal community composition.

Fungal genus	Frequency in rooms^a^
*Aspergillus* spp.	0.725
*Penicillium* spp.	0.549
*Cladosporium* spp.	0.510
*Trichoderma* spp.	0.255
*Chaetomium* spp.	0.157
*Epicoccum* spp.	0.137
*Alternaria* spp.	0.137
*Stachybotrys* spp.	0.098
*Fusarium* spp.	0.078

Fungal community composition observed on the gypsum board samples of 51 sampled rooms.

^a^ Example for *Aspergillus spp*.: Number of rooms observed = 37; 37/51 = 0.725

**Fig 3:**
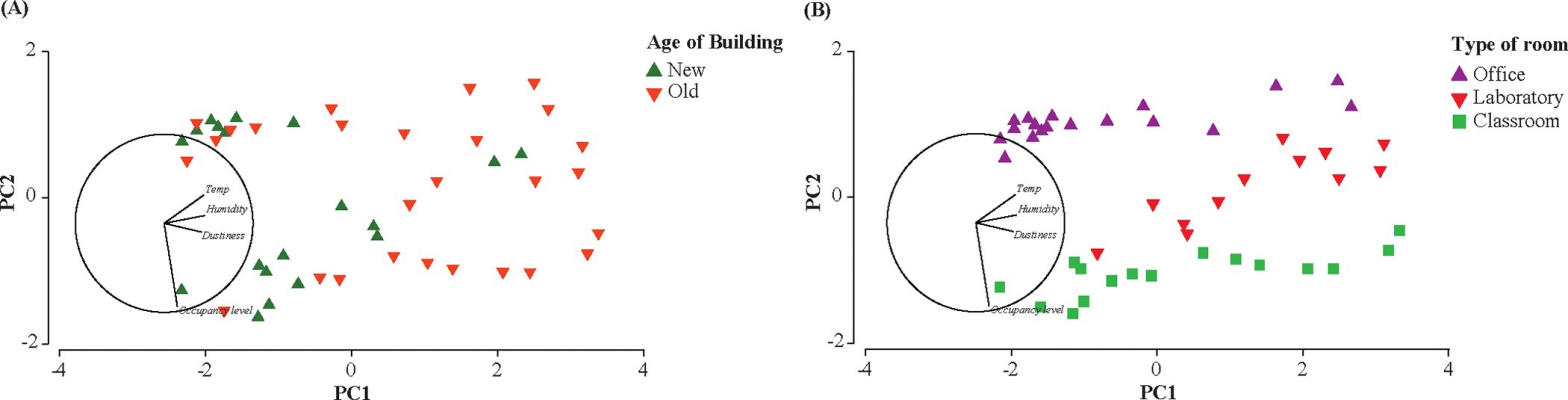
Principal component analysis (PCA) plot. PCA with the random variables of temperature, humidity, dustiness, and occupancy level and the controlled variables of (A) age of building and (B) type of room for the 51 rooms sampled. Axes are the principal component, PC1, and PC2, with loading values.

The random effects from uncontrolled factors that varied from room to room, including temperature, humidity, dustiness, and occupancy level were also analyzed. The plot in Fig 3 shows the interrelationship between these random (uncontrolled) variables and controlled variables of the age of building and type of room. The results of the clustering pattern (Fig 3A) via PCA supported the earlier analysis results on overall differences among the age of buildings. The older buildings compared to the new buildings are associated with having a higher temperature (22.5 ± 0.2°C; 22.3 ± 0.2°C), relative humidity (39.1 ± 1.6%; 37.8 ± 1.1%), and dustiness scores (2.2 ± 0.7; 1.9 ± 0.1), and a low or no association with occupancy level (Fig 3A). The latter was well justified by the fact the buildings had a similar number of classroom capacities and labs. A very distinguishable pattern among different types of rooms was also evident (Fig 3B). It can be induced that on average in each building, although no differences in temperature, humidity, and dustiness were found between the rooms (Fig 3B**)**, the labs had slightly higher values (mean temperature: 22.50 ± 0.2°C; mean relative humidity: 39.64 ± 2.1%; mean dustiness score: 2.75 ± 0.5), compared to the classrooms (22.36 ± 0.2°C; 38.53 ± 1.0%; 1.95 ± 0.6) and the offices (22.35 ± 0.2C; 37.9 ± 1.3%; 1.7 ± 0.8). In addition, the different types of rooms displayed a very significant association with the occupancy level, with classrooms as the most occupied space as expected (Fig 3B**)**.

### Detection of health hazardous fungal species

**Fig 4:**
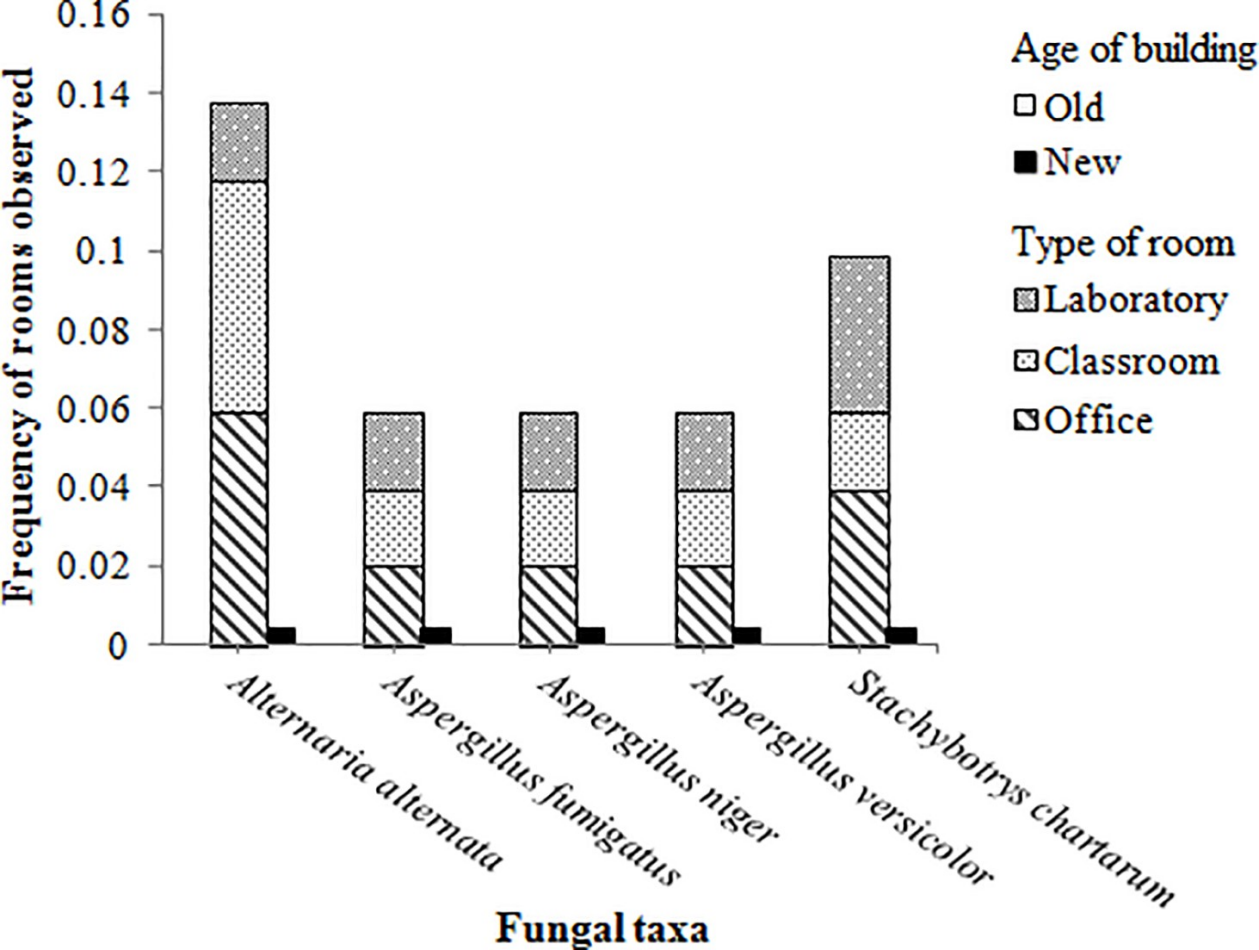
The fungal taxa present in rooms of the sampled buildings. No significant differences in fungal diversity were observed for the type of room and type of flooring. Significant differences in frequency of rooms were observed for each genus for the age of building, using chi-square test (p<0.05).

When comparing the fungal types observed on gypsum samples of older and newer buildings, there was a significantly higher proportion of rooms in older buildings with *A*. *alternata* (χ^2^(1) = 7.00, p = 0.008), *A*. *fumigatus* (χ^2^(1) = 4.00, p = 0.046), *A*. *niger* (χ^2^(1) = 4.00, p = 0.046), *A*. *versicolor* (χ^2^(1) = 4.00, p = 0.046), and *S*. *chartarum* (χ^2^(1) = 5.00, p = 0.025) (Fig 4). The trace of these five indoor fungal species hazardous to health was only found in dust samples from older buildings and on average more in the classrooms and labs that had higher dustiness levels (compare Fig 3 and Fig 4 for dustiness levels and variations in the presence of these fungal species across varying environments). No significant differences in the diversity of fungal genera were observed for the different types of rooms and flooring.

### Biodeterioration assessment: Weight loss and tensile testing

**Fig 5:**
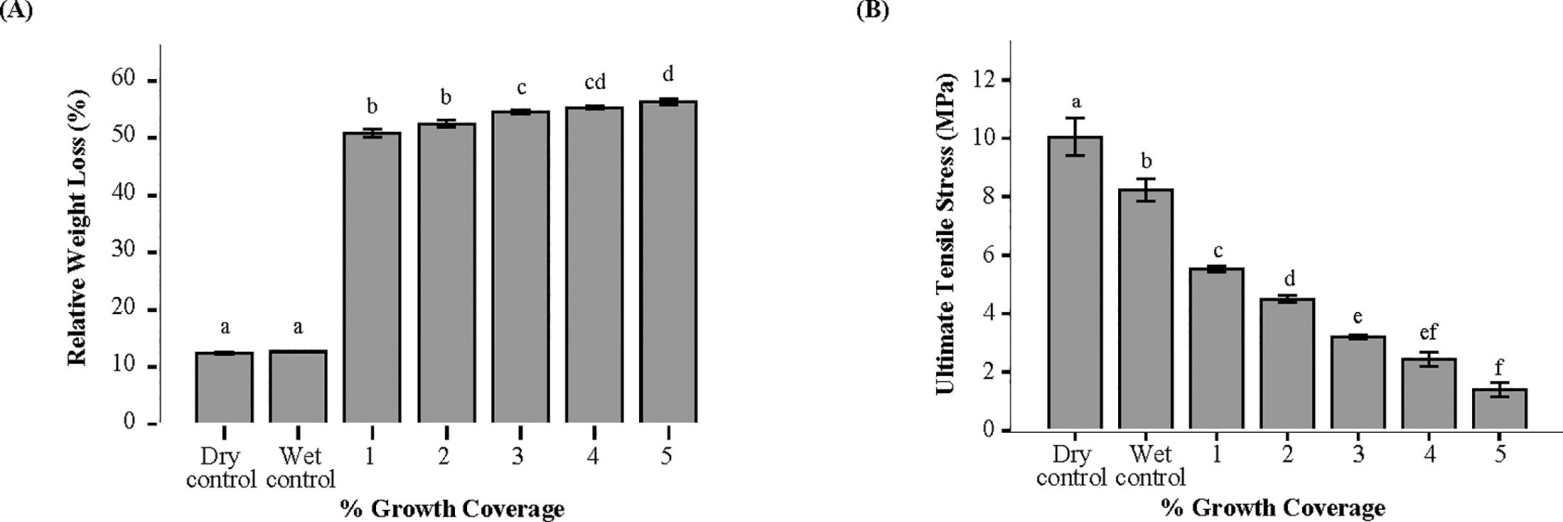
Biodeterioration assessment. (A) The relative weight loss (n = 35) over a one-week time period, and (B) ultimate tensile stress (n = 31) of gypsum board samples with varying ranges of % growth coverage of fungi (1–5). Dry control samples were not exposed to water or dust, while wet control samples were only exposed to water. Error bars indicate standard deviation. Columns with different capital letters for (A) indicate significant differences in relative weight loss using ANOVA and Tamhane's post hoc tests (F_6,35_ = 9810.2, p<0.05), and for (B) significant differences in tensile stress using ANOVA and Tamhane's post hoc tests (F_6,31_ = 396.3, p<0.05).

**Fig 6:**
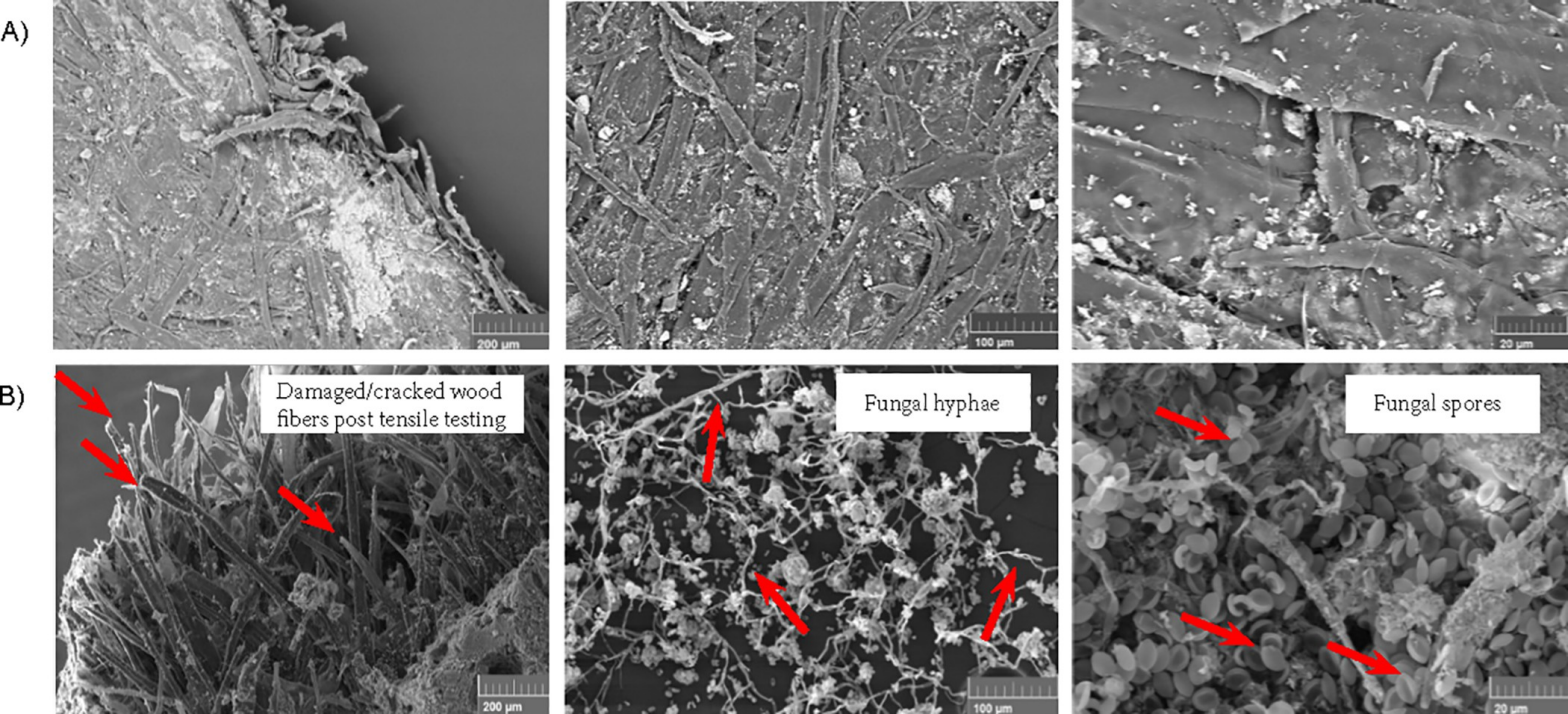
SEM micrographs of gypsum board samples. SEM of (A) the control pieces which were UV sterilized and not exposed to dust and high humidity conditions, and (B) the gypsum board pieces post tensile testing upon 4 weeks of exposure to dust and high humidity conditions.

Analysis of the dry weights of gypsum boards showed a significant weight loss over time, F_6,35_ = 9810.2, p<0.05 (Fig 5A). The wet control samples had an insignificant weight loss (12.7±0.10%) compared to the dry control groups (12.4±0.2%) (Fig 5A). The weight and physical property of the gypsum boards decreased as the % coverage of fungal growth increased (Fig 5A). The highest coverage range of fungal growth on the gypsum boards showed a 56.3±0.5% decrease in weight over time (Fig 5A). The physical (weight loss) and mechanical (tensile) properties of the gypsum boards were negatively correlated (r = -0.924, n = 21, p<0.01) (Fig 2B). In addition, the growth of fungi on the gypsum boards had a significant effect on the mechanical properties of the material, F_6,31_ = 396.3, p<0.05 (Fig 5B). The wet control samples had a 20% decrease in the tensile strength (8.2±0.4 MPa) when compared to the control groups (10.1±0.7 MPa) (Fig 5B). The tensile strength of the paper backing of the gypsum boards decreased as the % coverage of fungal growth on the gypsum boards increased (Fig 5B). The highest % coverage range of fungal growth on the gypsum boards showed an 86% decrease in the tensile strength. The gypsum board samples exposed to high humidity and fungi also led to the microstructural defects, as evident by cracks in the paper-backing, presence of powdered gypsum in the glass jars, and the gypsum and paperback interface separation. Fig 6A shows the SEM images of the control gypsum board samples, which were UV sterilized and contained no observed fungal growth on the surface of these materials. Fig 6B show SEM images post-tensile testing, with a clear visualization of spores and fungal hyphae. The fungi grew on the paper backing and the gypsum under ambient conditions (submerged in water and fungi from the collected dust samples). The damaged cracked fibers of cellulosic wood and fiber pull-out due to tensile testing of these samples is also seen to be much greater compared to the control samples (Fig 6).

## Discussion

The above tests examined the environmental conditions and factors that may lead to fungal growth on water-damaged building material of gypsum boards and observed the biodeteriorative capabilities of these organisms. Different building attributes and environmental conditions across the studied campus statistically influenced the fungal growth and its diversity on the conditioned gypsum boards. The findings show that older buildings on campus, on average, have a higher concentration and diversity of fungal taxa in the air, leading to higher mould coverage and biodeterioration on the gypsum boards. These observations are aligned with earlier findings in the literature [44, 55]. In particular, it can be attributed to the antiquated building material and techniques used for aged buildings, which have shown to hold higher moisture levels compared to new buildings [45], as also observed in this study where the older buildings had higher humidity and temperature on average. Some building materials such as solid lumber and bricks used in older buildings, however, have occasionally been reported to be advantageous compared to gypsum boards, since they have a high saturation threshold for moisture and hence can decrease condensation levels and be less susceptible to fungal growth [32]. The variation of the structural-frame is also a factor, since the concrete-frame of newer buildings have more CO_2_ and hydrocarbon emissions, while the steel-frame of older buildings have more volatile organic compounds (VOCs) and heavy metal emissions [56]. Older steel-framed buildings also have higher thermal conductivity and heat transfer, which can create higher temperatures in these buildings compared to newer buildings, which was supported by our results. Another related point is the atria existing in the older buildings, which contain plants that can also increase humidity and affect fungal diversity [45]. Some rooms within the aged buildings, depending on their functionalities, can also accumulate a higher dust level and lead to more indoor fungal spores and biodeterioration of gypsum boards. This can be due to the poorly insulated building envelopes, low airtightness, and less efficient heating ventilation and air conditioning (HVAC) systems, or may be due to the increased level of activities and number of occupants in those rooms [57]. Therefore, older buildings require more diligent monitoring and prompt remediation in cases of e.g. water leakage, flooding, and mould growth.

When observing the different types of rooms and their influence on fungal growth and diversity, classrooms with the highest occupancy level, showed a higher coverage and diversity compared to offices with low occupancy levels. People in high occupancy environments can create higher dust levels that enable microorganisms to grow [16], transport more bioaerosols from outdoor environments [58], and also increase humidity and temperature in indoor spaces [45]. The increased air velocity current by the movement of occupants can release more spores [59, 60]. Although labs had a medium occupancy level, they also had a higher fungal coverage and diversity response compared to the offices. The increased activity level of the laboratory’s occupants from conducted experiments and the use of materials and equipment can create high dust levels. Sources of fungi in labs could vary from field samples, animal testing, by-products purchased and stored materials that have accumulated fungal spores over time, to the experimenters’ labware. In addition to these effects, it is also important to note that labs and classrooms have HVAC systems that have a higher air change rate. This greater airflow can lead to an increased concentration of spores in these environments and as a result, increase fungal growth and diversity [61, 62]. Lab rooms in our study also contained windows that remained closed the majority of the time, which would lead to poor air circulation and increased humidity and temperature [63].

The factor of flooring type had no significant effect on the fungal growth and diversity observed on the tested gypsum board samples. However, it showed a favourable trend towards slightly increasing coverage and diversity of the indoor fungi, which is in support of the findings by Sharpe et al. [45] and Wani et al. [64]. In essence, carpets can provide a habitat to support fungal growth even without moisture damage and can increase indoor populations of fungi [65]. In contrast, Chew et al. (2003) found that although carpets contained a higher dust-borne fungal concentration than non-carpet rooms, it did not lead to higher levels of airborne fungi in the built environment [66].

Interestingly, concerning the fungal composition formed on the gypsum board samples, the most frequently occurring species in our study were consistent with earlier reports on the species composition of dust [59]. Namely, the results of our study displayed an abundant presence *of Aspergillus*, *Penicillium*, and *Cladiosporium* spp., and a lower abundance of *Alternaria*, *Stachybotrys*, *and Fusarium* spp. which is found in the atmosphere. This observation is consistent with previous findings [59, 67], which suggested that the most abundant species found produce small, light spores, in comparison to those that are less abundant and produce fewer, bigger, and heavier spores that are not easily airborne. The abundance of smaller spores can also be more problematic and cause allergenic responses when inhaled [59]. Our results suggested that environmental conditions and characteristics unique to each type of room within buildings can exert an influence on microbial communities available in that environment.

It is also important to note that this study focused on a fractional diversity of indoor fungal taxa since only viable fungi that were able to grow on the gypsum boards were tested and analyzed. From all of the observed fractional fungal taxa across the campus, *A*. *alternata*, *A*. *fumigatus*, *A*. *niger*, *A*. *versicolor*, and *S*. *chartarum* were found only in rooms of the older buildings. *Stachybotrys* spp. are most often associated with moist building conditions [27, 40], which possibly explains why their spores were most often found in older buildings that had higher humidity conditions. On the other hand, since *Stachybotrys* spp. produce spores in wet slimy heads and are not readily airborne [27, 67], their presence might be just as prevalent in newer buildings but not detectable. This could be due to other underlying factors that would aerosolize their spores in older buildings. No significant differences in fungal composition were observed between the different types of rooms and flooring conditions in the current case study, which may suggest that fungal spores are ubiquitous with respect to these two factors and disperse from outdoor sources [68]. The presence of *Alternaria* spp. in older buildings was also higher since they had higher humidity conditions and more rooms sampled with carpeted flooring, which can increase such fungi [64].

Fungal growth is a key agent of structural decay, which can change the structure of the paper backing of gypsum boards with a negative impact on their physical and mechanical properties. Biodeteroration can also affect the physical and mechanical properties of many other material substrates including wood, polyvinyl chloride (PVC), natural fibers, etc. [6, 47, 69]. The results of the study clearly indicate that deterioration by microorganisms can decrease both physical and mechanical properties of gypsum board material, as shown by the increased weight loss and decreased tensile strength of test samples. The wet control samples that contained no fungal growth, however, did not significantly affect the physical properties of gypsum boards, which shows that water coupled with the microbial growth is what mainly affects the physical properties of this material. In contrast, the wet control samples significantly affected the mechanical properties despite no fungal growth on the materials. The increased moisture exposure and fungi can create voids between the bondage and attachment of gypsum and paper, and as a consequence lead to extensive de-bonding and reduction of the material load carrying capacity. This is supported by a study from the Canada Housing and Mortgage Corporation that found a 0–2% decrease in the flexural strength and an increase in moisture content by 5% caused gypsum panels to crumble [70]. The porosity of the gypsum can also decrease the mechanical properties of gypsum board materials [71] since they have high water holding capabilities and lead to fungal growth [43]. This high moisture retention can additionally lead to the aggregation of gypsum crystals which reduces contact with neighbouring crystals and lowers the structural efficiency of the material [71]. Structural properties of gypsum board materials are important for building designers, as they are frequently used in construction, and thus evaluating how microorganisms may affect these materials performance is crucial. In particular, further research can aid manufacturers, building designers, and construction workers to i) uncover novel materials that are less susceptible to fungal growth, ii) to optimize the design of built environments via energy efficiency, control of temperature, and humidity conditions, iii) increase the application of mould-resistant gypsum boards, and iv) identify new feasible and efficient remediation techniques.

When observing the mould on the gypsum board samples for health hazardous fungi, five fungal species (*A*. *niger*, *A*. *fumigatus*, *A*. *versicolor*, *A*. *alternata*, and *S*. *chartarum*) that are known to be human pathogens were detected only in older buildings. It is known that most fungal species present in indoor environments come from outdoor sources [23]. Although only a fraction of fungal species present in outdoor environments has been detected in indoor environments [67], numerous health effects have been attributed to these indoor fungi [27, 52, 59]. The presence of these pathogenic fungal species can lead to mycotoxins (such as ochratoxin, gliotoxin, sterigmatocystin, and trichothecenes) and VOCs, which can increase the prevalence of diseases [25, 64]. Exposure to the allergenic fungi *A*. *alternata* can induce skin and pulmonary infections [40, 45]. *S*. *chartarum*, commonly known as black mould, can also lead to sick building syndrome (SBS), which can lead to unpleasant odors in indoor environments and symptoms such as headaches, dizziness, fatigue, and difficulty concentrating [3, 29]. As reviewed in the introduction, indoor mould growth can also have a major economic impact and increase health care costs, building repair costs, and business costs [72, 73]. Thus, human exposure assessment and environmental evaluations, next to structural performance assessments, are deemed an essential component of healthy building design, especially after events such as major water damage or flooding.

## Concluding remarks

Although there have been a number of studies on indoor factors affecting fungal growth on different building materials, the underlying mechanisms of such association with building characteristics are not well understood and require further experimental investigations. The results of this campus-wide case study suggested that older buildings and their laboratories and classrooms would require more diligent monitoring, proper ventilation, and frequent cleaning (e.g., as a proactive measure for potential water system damage, flooding, natural disasters, etc.). The type of flooring (presence or absence of carpet) was seen to be a second order effect influencing the level of fungal growth and diversity on building materials. The role of fungi in the material structural deterioration was strongly supported by the performed tests on gypsum boards, which demonstrated that different fungal microorganisms can affect both physical and mechanical properties, as well the microstructural integrity of the gypsum boards over time. It is important to note that this study used a simulated extreme case of high humidity conditions and although these fungal species were found to be present in the indoor environment, exposure levels of the airborne microorganisms were not measured and should be considered for future studies. It is also important to note that the significant factors identified are only a fraction of those available that can affect indoor microbiota. Further research is recommended to expand knowledge on the interactions between the deterioration of other types of engineered materials and built environmental biota as a function of building characteristics. Such new insights can eventually enable optimal design of highly microbial-resistant building materials while decreasing long-term economic losses and occupant health concerns.

## Supporting information

S1 Fig The experimental set up of drywall samples. 5 cm × 8 cm drywall pieces (n = 102) were submerged vertically in water containing the collected dust samples from each room (n = 51). Mould growth was observed on the samples over time.
(TIFF)

S1 File The raw data used for analysis. The data utilized for figure creation and data analysis in this study.
(XLSX)

## References

[1] Flannigan B, Samson RA, Miller JD. Microorganisms in home and indoor work environments: Diversity, health impacts, investigation and control 2nd ed. Boca Raton, FL: CRC Press; 2011.

[2] Nielsen KF, Nielsen PA, Thrane U, Larsen TO, Gravesen S. Production of mycotoxins on artificially inoculated building materials. Int Biodeterior Biodegradation. 1998;42(1):9–16.

[3] Betancourt DA, Krebs K, Moore SA, Martin SM. Microbial volatile organic compound emissions from *Stachybotrys chartarum* growing on gypsum wallboard and ceiling tile. BMC Microbiology. 2013;13(1):283. 24308451 10.1186/1471-2180-13-283 PMC4234204

[4] Genuis SJ. Clinical medicine and the budding science of indoor mold exposure. Eur J Intern Med. 2007;18(7):516–23. 10.1016/j.ejim.2007.05.005 17967332

[5] Portnoy JM, Barnes CS, Kennedy K. Sampling for indoor fungi. J Allergy Clin Immunol. 2004;113(2):189–98. 10.1016/j.jaci.2003.11.021 14767427

[6] Webb JS, Nixon M, Eastwood IM, Greenhalgh M, Robson GD, Handley PS. Fungal colonization and biodeterioration of plasticized polyvinyl chloride. J App Environ Microbiol. 2000;66(8):3194–200. 10.1128/aem.66.8.3194-3200.2000 PMC92133 10919769

[7] Households and the environment Statistics Canada; 2015 [Available from: https://www150.statcan.gc.ca/n1/pub/11-526-x/2011001/part-partie1-eng.htm.

[8] Where the mould grows.: Drews, K; 2008 [Available from: https://www.theglobeandmail.com/news/national/where-the-mould-grows/article22502094/.

[9] Rayner ADM, Boddy L. Fungal decomposition of wood: Its biology and ecology. New York: Wiley; 1988.

[10] Sanchez-Silva M, Rosowsky DV. Biodeterioration of construction materials: State of the art and future challenges. J Mater Civil Eng. 2008;20(5):352–65.

[11] Cost to remove drywall: Remodeling expense; 2018 [Available from: https://www.remodelingexpense.com/services/cost-to-remove-drywall/.

[12] Install or hang drywall: Home Advisor; 2018 [Available from: https://www.homeadvisor.com/cost/walls-and-ceilings/drywall-installation-hanging/.

[13] Hanna J, Taggart P. Balcony collapses during Irish students’ party in Berkeley, killing 6; 2015 [Available from: https://www.cnn.com/2015/06/16/us/california-balcony-collapse/index.html.

[14] Fromme H, Gareis M, Völkel W, Gottschalk C. Overall internal exposure to mycotoxins and their occurrence in occupational and residential settings–an overview. Int J of Hyg Environ Health. 2016;219(2):143–65. 26725999 10.1016/j.ijheh.2015.11.004

[15] Andersen B, Nielsen KF, Jarvis BB. Characterization of Stachybotrys from water-damaged buildings based on morphology, growth, and metabolite production. Mycologia. 2002;94:392–403. 21156510

[16] Brown DW, Yu J, Kelkar HS, Fernandes M, Nesbitt TC, Keller NP, et al Twenty-five coregulated transcripts define a sterigmatocystin gene cluster in aspergillus nidulans. Proc Natl Acad Sci U S A. 1996;93(4):1418–22. 10.1073/pnas.93.4.1418 8643646 PMC39953

[17] Hope J. A review of the mechanism of injury and treatment approaches for illness resulting from exposure to water-damaged buildings, mold, and mycotoxins. Scientific World Journal. 2013;2013:767482–20. 10.1155/2013/767482 23710148 PMC3654247

[18] Ratnaseelan AM, Tsilioni I, Theoharides TC. Effects of mycotoxins on neuropsychiatric symptoms and immune processes. Clin Ther. 2018;40(6):903–17. 10.1016/j.clinthera.2018.05.004 29880330

[19] Karunasena E, Larrañaga MD, Simoni JS, Douglas DR, Straus DC. Building-associated neurological damage modeled in human cells: A mechanism of neurotoxic effects by exposure to mycotoxins in the indoor environment. Mycopathologia. 2010;170(6): 377–90. 10.1007/s11046-010-9330-5 20549560

[20] Tischer C, Tiesler C, Standl M, Gehring U, Wijga A, Melen E, et al Dampness and mould on respiratory health–A longitudinal approach. Results from the MeDALL study. Eur Respir J. 2016;48(suppl 60):OA3319.

[21] Mudarri D, Fisk WJ. Public health and economic impact of dampness and mold. Indoor Air. 2007;17(3):226–35. 10.1111/j.1600-0668.2007.00474.x 17542835

[22] Baxi SN, Portnoy JM, Larenas-Linnemann D, Phipatanakul W. Exposure and health effects of fungi on humans. J Allergy Clin Immunol Pract. 2016;4(3):396–404. 10.1016/j.jaip.2016.01.008 26947460 PMC4861659

[23] Adams R, Miletto M, Taylor J, Bruns T. Dispersal in microbes: Fungi in indoor air are dominated by outdoor air and show dispersal limitation at short distances. Isme Journal. 2013;7(7):1262–73. 10.1038/ismej.2013.28 23426013 PMC3695294

[24] Gadd GM. Mycotransformation of organic and inorganic substrates. Mycologist. 2004;18(2):60–70.

[25] Nielsen KF, Holm G, Uttrup LP, Nielsen PA. Mould growth on building materials under low water activities. Influence of humidity and temperature on fungal growth and secondary metabolism. Int Biodeterior Biodegradation. 2004;54(4):325–36.

[26] Haleem Khan AA, Mohan Karuppayil S. Fungal pollution of indoor environments and its management. Saudi J Biol Sci. 2012;19(4):405–26. 10.1016/j.sjbs.2012.06.002 23961203 PMC3730554

[27] Andersen B, Frisvad JC, Sondergaard I, Rasmussen IS, Larsen LS. Associations between fungal species and water-damaged building materials. Appl Environ Microbiol 2011;77(12):4180–8. 10.1128/AEM.02513-10 21531835 PMC3131638

[28] Hyvärinen A, Meklin T, Vepsäläinen A, Nevalainen A. Fungi and actinobacteria in moisture-damaged building materials—concentrations and diversity. Int Biodeterior Biodegradation. 2002;49(1):27–37.

[29] Karunasena E, Markham N, Brasel T, Cooley JD, Straus DC. Evaluation of fungal growth on cellulose-containing and inorganic ceiling tile. Mycopathologia. 2001;150(2):91–5. 11407495 10.1023/a:1010920611811

[30] Flannigan B, Samson RA, Miller JD. Microorganisms in home and indoor work environments: Diversity, health impacts, investigation and control London: Taylor & Francis; 2001.

[31] Mankowski M, Morrell JJ. Patterns of fungal attack in wood-plastic composites following exposure in a soil block test. Wood Fiber Sci. 2000;32:340–5.

[32] Yan L, Morrell JJ. Mold and decay resistance of thermally modified douglas-fir heartwood. Forest Products J. 2015;65(5–6):272–7.

[33] McMullin DR, Sumarah MW, Miller JD. Chaetoglobosins and azaphilones produced by Canadian strains of *Chaetomium globosum* isolated from the indoor environment. Mycotoxin Res. 2013;29(1):47–54. 10.1007/s12550-012-0144-9 23334724

[34] Pasanen A, Pasanen P, Korpi A, Kalliokoski P. Growth and volatile metabolite production of *Aspergillus versicolor* in house dust. Environment International 1997;23(4):425–32.

[35] Foarde K, Berry M. Comparison of biocontaminant levels associated with hard vs. carpet floors in nonproblem schools: Results of a year long study. J Expo Sci Environ Epidemiol. 2004;14(S1):S41–S8. 10.1038/sj.jea.7500357 15118744

[36] Hicks JB, Lu ET, De Guzman R, Weingart M. Fungal types and concentrations from settled dust in normal residences. J Occup Environ Hyg. 2005;2(10):481–92. 10.1080/15459620500252860 16109703

[37] Phelka AD, Finley BL. Potential health hazards associated with exposures to asbestos-containing drywall accessory products: A state-of-the-science assessment. Crit Rev Toxicol. 2012;42(1):1–27. 10.3109/10408444.2011.613067 22044019

[38] Kolarkar P, Mahendran M. Experimental studies of gypsum plasterboards and composite panels under fire conditions. Fire Mater. 2014;38(1):13–35.

[39] Ghazi Wakili K, Koebel M, Glaettli T, Hofer M. Thermal conductivity of gypsum boards beyond dehydration temperature. Fire Mater. 2015;39(1):85–94.

[40] Grant C, Hunter CA, Flannigan B, Bravery AF. The moisture requirements of moulds isolated from domestic dwellings. Int Biodeterior Biodegradation. 1989;25(4):259–84.

[41] Vacher S, Hernandez C, Bärtschi C, Poussereau N. Impact of paint and wall-paper on mould growth on plasterboards and aluminum. Build Environ. 2010;45(4):916–21.

[42] Verdier T, Coutand M, Bertron A, Roques C. A review of indoor microbial growth across building materials and sampling and analysis methods. Build Environ. 2014;80:136–49.

[43] Dedesko S, Siegel JA. Moisture parameters and fungal communities associated with gypsum drywall in buildings. Microbiome. 2015;3:71 10.1186/s40168-015-0137-y 26642923 PMC4672539

[44] Sivasubramani SK, Niemeier RT, Reponen T, Grinshpun SA. Assessment of the aerosolization potential for fungal spores in moldy homes. Indoor Air. 2004;14(6):405–12. 10.1111/j.1600-0668.2004.00262.x 15500633

[45] Sharpe R, Thornton CR, Osborne NJ. Modifiable factors governing indoor fungal diversity and risk of asthma. Clin Exp Allergy. 2014;44(5):631–41. 10.1111/cea.12281 24471926

[46] Krause M, Geer W, Swenson L, Fallah P, Robbins C. Controlled study of mold growth and cleaning procedure on treated and untreated wet gypsum wallboard in an indoor environment. J Occup Environ Hyg. 2006;3(8):435–41. 10.1080/15459620600798663 16862714

[47] Pekhtasheva E, Neverov A, Kubica S, Zaikov G. Biodegradation and biodeterioration of some natural polymers. Chem Technol. 2012;6(3).

[48] Viitanen H, Vinha J, Salminen K, Ojanen T, Peuhkuri R, Paajanen L, et al Moisture and bio-deterioration risk of building materials and structures. J Build Phys. 2010;33(3):201–24.

[49] Andersen B, Dosen I, Lewinska AM, Nielsen KF. Pre-contamination of new gypsum wallboard with potentially harmful fungal species. Indoor Air. 2017;27(1):6–12. 10.1111/ina.12298 26970063

[50] Adams RI, Tian Y, Taylor JW, Bruns TD, Hyvarinen A, Taubel M. Passive dust collectors for assessing airborne microbial material. Microbiome. 2015;3:46 10.1186/s40168-015-0112-7 26434807 PMC4593205

[51] Area measurement of a complex object: ImageJ; 2018 [Available from: https://imagej.nih.gov/ij/docs/examples/index.html.

[52] Mihinova D, Pieckova E. Moldy buildings, health of their occupants and fungal prevention. Bratsil Med J. 2012;113(05):314–8. 22616593

[53] Haugland R, Vesper S, inventors; US Environmental Protection Agency, assignee. Method of identifying and quantifying specific fungi and bacteria. US2002.

[54] Quinn GP, Keough MJ. Experimental design and data analysis for biologists New York: Cambridge University Press; 2002.

[55] Dacarro C, Picco AM, Grisoli P, Rodolfi M. Determination of aerial microbiological contamination in scholastic sports environments. J Appl Microbiol 2003;95(5):904–12. 14633018 10.1046/j.1365-2672.2003.02044.x

[56] Guggemos AA, Horvath A. Comparison of environmental effects of steel- and concrete-framed buildings. J Infrastruct Syst. 2005;11(2):93–101.

[57] Zhao D, Azimi P, Stephens B. Evaluating the long-term health and economic impacts of central residential air filtration for reducing premature mortality associated with indoor fine particulate matter (PM2.5) of outdoor origin. Int J Environ Res Public Health. 2015;12(7):8448–79. 10.3390/ijerph120708448 26197328 PMC4515730

[58] Muilenberg ML. The outdoor aerosol CRC Press I, editor. Boca Raton, Florida 1995.

[59] Cabral JS. Can we use indoor fungi as bioindicators of indoor air quality? Historical perspectives and open questions. Sci Total Environ. 2010;408:4285–95. 10.1016/j.scitotenv.2010.07.005 20655574

[60] Frankel M, Hansen EW, Madsen AM. Effect of relative humidity on aerosolization and total inflammatory potential of fungal particles from dust-inoculated gypsum boards. Indoor Air. 2014;24:16–28. 10.1111/ina.12055 23750665

[61] Rogers CA. Indoor fungal exposure. Immunol Allergy Clin North Am. 2003;23:501–18. 14524388 10.1016/s0889-8561(03)00061-4

[62] Dannemiller KC, Gent JF, Leaderer BP, Peccia J. Influence of housing characteristics on bacterial and fungal communities in homes of asthmatic children. Indoor Air. 2016;26:179–92. 10.1111/ina.12205 25833176 PMC4591094

[63] Jamriska M, Morawska L, Clarke BA. Effect of ventilation and filtration on submicrometer particles in an indoor environment. Indoor Air. 2000;10: 19–26. 10842457 10.1034/j.1600-0668.2000.010001019.x

[64] Wani KA, Mamta K, Khan TA, Lone R. Fungal contamination of carpet industry in Gwalior Madhya Pradesh (India). Indoor Built Environ. 2014;23(5):724–9.

[65] Kemp PC, Neumeister-Kemp HG, Koch C, Lysek G, Murray F. Determining the growth and vitality of microorganisms in carpets and mattresses in non-problem dwellings by measuring CO2 released during respiration. Indoor Built Environ. 2002;11:214–20.

[66] Chew GL, Rogers C, Burge HA, Muilenberg ML, Gold DR. Dustborne and airborne fungal propagules represent a different spectrum of fungi with differing relations to home characteristics. Allergy. 2003;58(13–20). 10.1034/j.1398-9995.2003.00013.x 12580801

[67] Adan OCG, Samson RA. Fundamentals of mold growth in indoor environments and strategies for healthy living. Publisher WA, editor. The Netherlands2011.

[68] Dumbrell AJ, Nelson M, Helgason T, Dytham C, Fitter AH. Relative roles of niche and neutral processes in structuring a soil microbial community. ISME J. 2010;4(3):337–45. 10.1038/ismej.2009.122 19924158

[69] Crawford B, Pakpour S, Kazemian N, Klironomos J, Stoeffler K, Rho D, et al Effect of fungal deterioration on physical and mechanical properties of hemp and flax natural fiber composites. Materials. 2017;10(11):1252. 10.3390/ma10111252 PMC5706199 29088118

[70] Canada Housing and Mortgage Association. Relationship between moisture content and mechanical properties of gypsum sheathing 2007 [Available from: http://publications.gc.ca/collections/collection_2011/schl-cmhc/nh18-1-2/NH18-1-2-173-2005-eng.pdf.

[71] Chen A, Sucech S, Faber KT. A hierarchical study of the mechanical properties of gypsum. J Mater Sci. 2010;45:4444–53.

[72] Fisk WS, Lei-Gomez Q, Mendell MJ. Meta-analyses of the associations of respiratory health effects with dampness and mold in homes. Indoor Air. 2007;17(4):284–96. 10.1111/j.1600-0668.2007.00475.x 17661925

[73] Mudarri DH. Valuing the economic costs of allergic rhinitis, acute bronchitis, and asthma from exposure to indoor dampness and mold in the US. J Environ Public Health. 2016;2386596:1–12. 10.1155/2016/2386596 PMC4903120 27313630

