# Supplementary figures and images for: Environmental factors influencing fungal growth on gypsum boards and their structural biodeterioration: A university campus case study
Source: PLoS One. 2019 Aug 2;14(8):e0220556. doi: 10.1371/journal.pone.0220556 (PMC6677307; doi:10.1371/journal.pone.0220556)

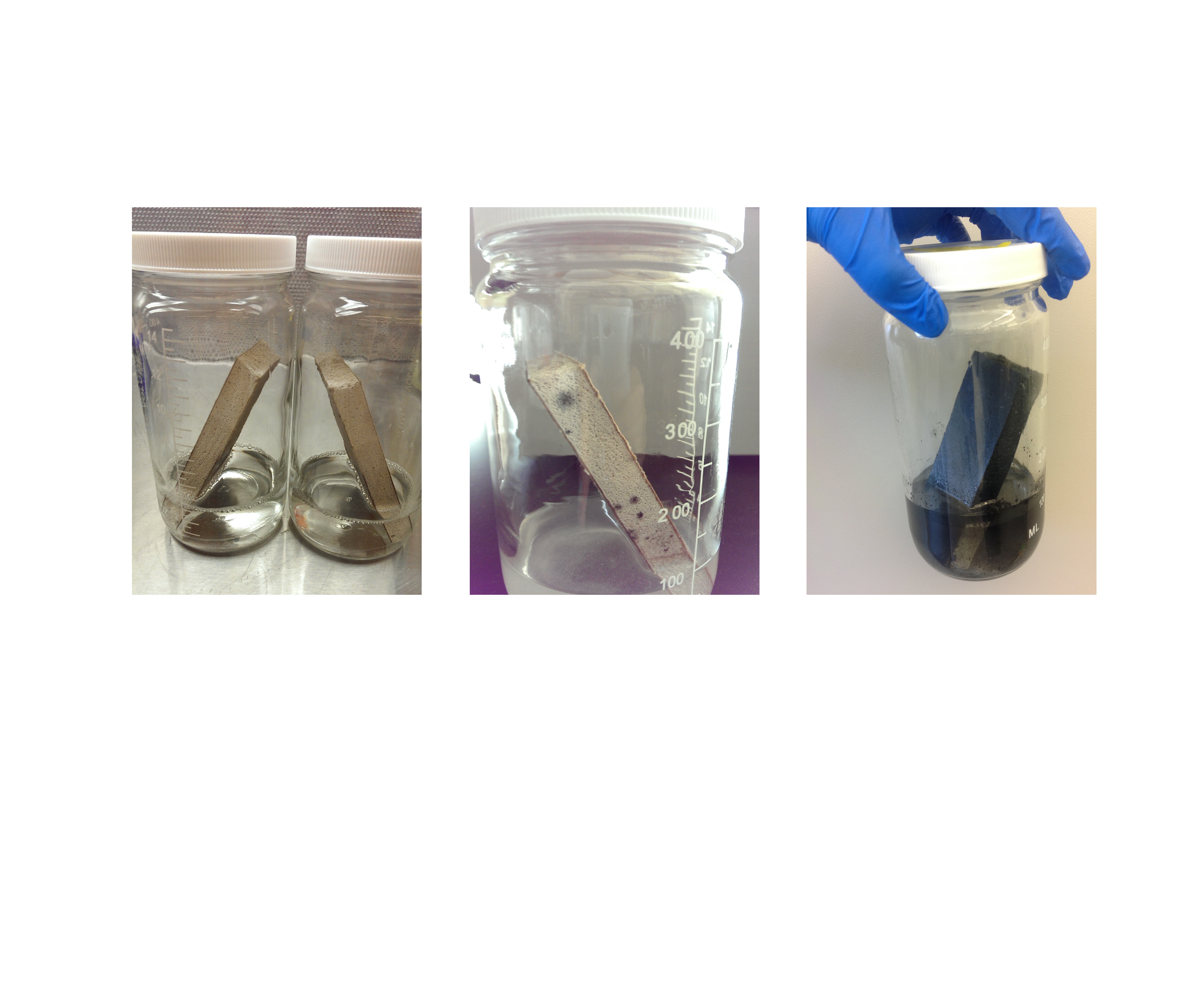

Supplement: S1 Fig — 5 cm × 8 cm drywall pieces (n = 102) were submerged vertically in water containing the collected dust samples from each room (n = 51). Mould growth was observed on the samples over time. (TIFF) [file pone.0220556.s001.tiff]
